# Unraveling lignin degradation in fibre cement via multidimensional fluorometry

**DOI:** 10.1038/s41598-023-35560-3

**Published:** 2023-05-24

**Authors:** Mahfuzul Hoque, Saeid Kamal, Sreenath Raghunath, E. Johan Foster

**Affiliations:** 1 Department of Chemical and Biological Engineering, Pulp and Paper Centre, The University of British Columbia, 2385 East Mall, British Columbia, V6T 1Z4 Canada; 2 Laboratory for Advanced Spectroscopy and Imaging Research (LASIR), Department of Chemistry, The University of British Columbia, 2036 Main Mall, Vancouver, BC V6T 1Z1 Canada; 3 Bioproducts Institute, 2385 East Mall, Vancouver, BC V6T 1Z4 Canada

**Keywords:** Chemistry, Engineering, Materials science

## Abstract

Pulp fibre reinforced cement (fibre cement) has the potential to become a forerunner in mitigating the carbon dioxide (CO2) footprint of non-structural materials for residential and commercial structures. However, one of the significant bottlenecks in fibre cement is its poor chemical stability in the alkaline cement matrix. To date, probing the health of pulp fibre in cement is lengthy and laborious, requiring mechanical and chemical separations. In this study, we have demonstrated that it is possible to understand the chemical interactions at the fibre-cement interfaces by tracking lignin in a solid state without using any additional chemicals. For the first time, multidimensional fluorometry is employed for the rapid assessment of the structural change (degradation) of lignin in fibre cement as an indicator of pulp fibre health; providing an excellent platform for the germination of resilient fibre cement with high content of natural lignocellulosic fibre.

Fibre cement is a fibre (natural/synthetic) reinforced composite^1^, where pulp fibre is predominantly employed owing to their abundance of raw materials (~ 145 × 10^6^ metric tons, chemical pulp production (2018))^2^, and low production cost (e. g., one third or less of glass fibres)^3^. Besides their mechanical prowess (high aspect ratio)^3–5^, it renders better termite resistance, and pest (i.e., woodpeckers) resistance^6,7^ than the wood particles/sawdust reinforced cement. Importantly, the low-density and low conductivity of pulp fibre affords the composite to be lightweight and insulative building materials; an ideal “low carbon” component that contributes to a high score in Leadership in Energy and Environmental Design (LEED) certification of commercial and residential buildings^6,7^.

Cement alkalinity (pH: 12.0–13.5) has a strong degradation effect on natural fibres^8,9^ analogous with the chemical pulping process (e.g., soda pulping), which is ubiquitous in the pulp and paper industry for the treatment of wood to remove lignin, and hemicellulose^10^. Note that lignocellulosic pulp fibre (hereinafter labeled as Ligno-Pulp) has several discernible features, for instance, long length (vital for tensile strength), thin and collapsible walls (creates a much better fibre network), low content of fines (affects pot life), and low coarseness (reduces the weight of the composite). Therefore, applications of Ligno-Pulp﻿ fibre in engineered composites^11,12^ including reinforced cement and mortar have been well-documented in the past 10 years^13^. However, to date, one of the *Achilles heels* of Ligno-Pulp is their poor alkaline stability, which necessitates modification (i.e., pH management) of the fabrication process of fibre cement. Note that longevity (> 20 yrs) of fibre cement is quintessential for circular economy and fibre cement will fail to maintain fibre-matrix bonding (loss of mechanical integrity) if the severity of cement matrix on Ligno-pulp becomes high. Most studies on durability (mechanical stability) of fibre cement are based on lengthy accelerated aging experiments (e.g., wet-dry cycles), which are highly time-consuming, and taxing on labor and materials. On the contrary, spectroscopic and/or microscopic characterization techniques^14^ are rapid, and non-destructive, and can provide direct evidence about the phenomena that occur at the fibre–cement interface even at the preliminary stages of curing (i.e., 7d).

To this end, fluorescence spectroscopy could be a potential platform technology, which boasts high throughput, and high sensitivity. In multidimensional fluorometry, excitation-emission map (EEM) and total synchronous fluorescence spectroscopy (TSFS) have become powerful characterization tools for complex materials (and/or mixture) and process monitoring, such as lignocellulosic fibres^15^ and depolymerization of technical lignin^16^. Lignin, a phenolic biopolymer composed of three major monolignols, *p*-coumaryl alcohol, Coniferyl alcohol, and Sinapyl alcohol joined via various inter-unit linkages (e.g., aryl ether unit (*β*–O–4)^17^—upon their inclusion into lignin, these alcohols are referred to as H-, (hydroxyphenyl); G-, (Guaiacyl); and S-, (Syringyl) lignin units, respectively^18,19^. Based on their biomass origin, lignin can be termed G-lignin (softwood; contains a small amount of H-lignin), GS-lignin (hardwood), and GSH-lignin (herbaceous plant)^20^. Importantly, variation (side chains and functional groups) in the lignin’s structure, which could be either natural and/or process induced has a profound effect on its autofluorescence characteristics (intensity and emission maximum)^21^ Now, the presence of unsaturated functional group bearing chemical motifs, such as phenylcoumarane, stilbene, and biphenyl chromophoric groups are potentially responsible for the different coloration of pulp fibre based on their production process i.e., yellow coloration of chemo-thermomechanical pulp (CTMP) pertinent to ∝, β-unsaturated aldehydes, and quinonoid structures, to name a few^22^.

Thus, autofluorescence of lignin has the potential to be employed as a label-free tag, which is yet to be exploited in composite materials, i.e., fibre cement. And being cognizant of the current research gap, in this study fibre cement (composite) was prepared without adding any fluorescent-tagged superplasticizer^23^ and to depict fibre-matrix interactions no additional laborious post-processing (as mentioned in time)^24^ treatments were necessary. By leveraging lignin autofluorescence in Ligno-Pulp, with appropriate (chemical-free) methodology, we have outlined (Fig. 1) how we can track pulp fibre “health” in fibre cement.

**Figure 1:**
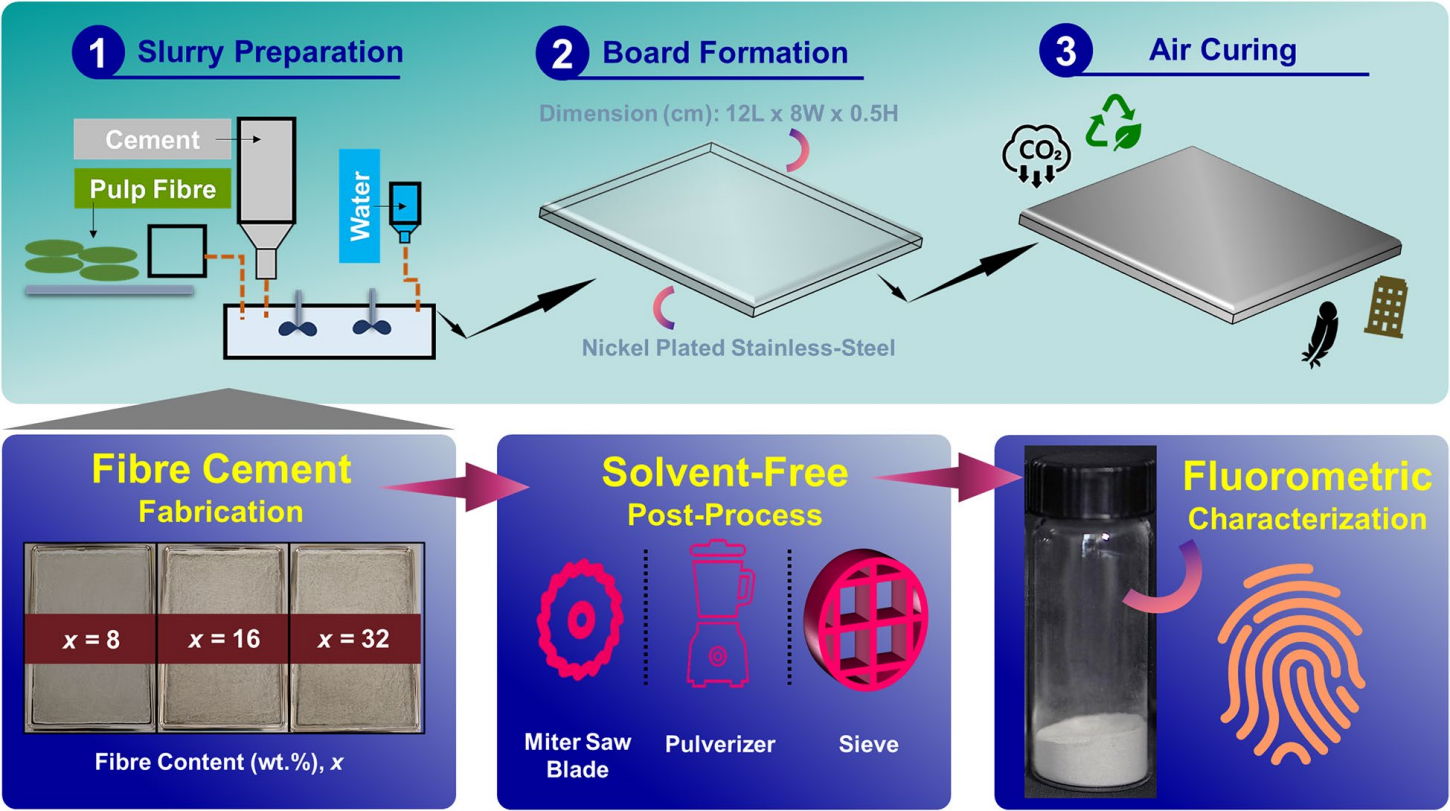
The fabrication process and fluorometric characterization of fibre cement. A representative outline of fibre cement fabrication and the sequence of post-processing steps (solvent-free) of the fabricated composite. The “*fingerprint*” cartoon (at bottom right side) symbolizes the technical feature of multidimensional fluorometry^25^. For post-processing and characterization details, see materials and methods section in the manuscript and the supplementary file.

Typical multidimensional fluorometry involves three parameters, which are excitation wavelength (*λ*_ex_), emission wavelength (*λ*_em_), and emission intensity (*I*_em_), and thereby result in an excitation-emission data matrix/map (EEM)^26^ — regarded as system fingerprints encoding valuable information related to the fluorescent probes and their interactions with the microenvironments^27,28^. On the other hand, in total synchronous fluorescence spectroscopy (TSFS), by simultaneous scanning of both the excitation and emission wavelengths by pre-setting a range of offset wavelengths depending on the material. Like EEM, it also involves three parameters, which are *λ*_ex_, offset emission wavelength (Δ*λ*_em_), and emission intensity (*I*_em_) and it is effective in understanding coordination polymer^29^, metal–organic frameworks (MOFs)^30^, crude oils, complex biological materials^31–33^, and conjugated compounds owing to its higher selectivity than EEM.

In this study, we have performed in-depth multidimensional fluorometric characterization, such as, EEM and TSFS to provide an understanding of the chemical interaction between lignin (as a component in pulp fibre) and cement hydration products (formed when cement particles contact water), which is also verified by additional microscopic and spectroscopic techniques, such as, X-ray fluorescence (XRF), powder X-ray diffraction (XRD), and energy dispersive spectroscopy (EDS). Also, we have provided a detail fluorometry-based qualitative and quantitative methodology to track “fibre-health” (indicator: lignin) in fibre cement.

## Results and discussion

The results and discussion section are structured in three parts, which are as follows:

- In the first segment, solid state EEM (emission map) was applied on the pulp fibre (Ligno-Pulp) and other lignocellulosic reference materials (Table 1) — a fluorescence “fingerprint” database was developed to establish a baseline for the fluorometric characterization of lignin in a complex matrix, i.e., cement. Then, the concept of “*photo-selective*” excitation was introduced (based on EEM analysis) to decouple the fluorescence of lignin from cellulosic components; then, it was utilized to visualize lignin in Ligno-Pulp and fibre cement (FC-Lab) via two-photon (2P) microscopy.
- Next, in the second segment, solid state EEM (emission map) was applied on pulp fibre (Ligno-Pulp) incorporated in cement matrix (Table 1) — a quantitative method was proposed to monitor lignin degradation (solid-state) in fibre cement based on chemical interaction between lignin and cement hydration products. Then, SEM–EDS, XRF, and XRD characterizations were performed to support the findings from EEM.
- Finally, in the third segment, solid state TSFS (synchronous map) was applied on pulp fibre (Ligno-Pulp), lignin model compound, and fibre cement (Table 1) — a synchronous fluorescence-based approach was presented to monitor the breakdown of lignin’s of conjugated polymeric structure into monomeric components in fibre cement.

﻿The list of materials used for fluorometric characterization is given below:

**Table 1:** List of raw materials and fibre cement (FC) composite. In the case of W-Ligno-Pulp, “W” stands for waste pulp fibre from pulp and paper mills. Ligno-CNF refers to lignocellulosic nanofibrils, which were prepared per the procedure described by Imani and others^34^.

Raw materials (*x* = 100)		Fibre cement (FC)	
Lignocellulosic	Ligno-Pulp	Lab-made	FC-Lab (*x* = 8)
	Ligno-CNF		FC-Lab (*x* = 16)
	Ligno-Xylan		FC-Lab (*x* = 32)
	W-Ligno-Pulp		
Cellulosic	Bleached-Pulp	Commercial	FC-Commercial
	∝ -Cellulose		
Lignin model compound	Guaiacylglycerol-β-guaiacyl ether		

### EEM (Emission map) Characterization of Lignocellulosic and Cellulosic Materials

**Figure 2:**
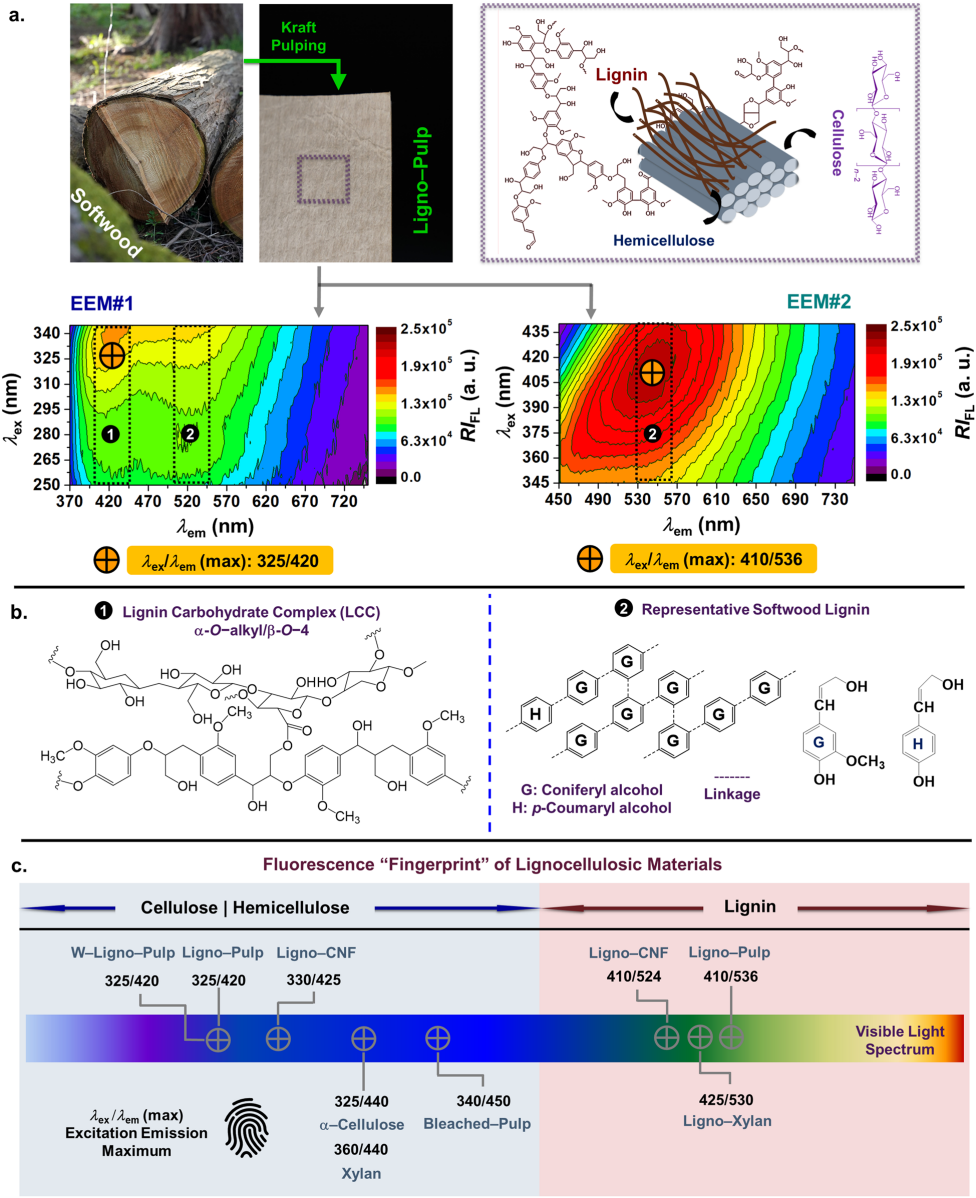
EEM (emission map) characterization of lignocellulosic materials. (**a**) (Top) Digital photograph and structural components of softwood derived unbleached Kraft pulp (labeled as Ligno-Pulp). Emission maps of Ligno-Pulp under (bottom left, EEM#1) shorter and (bottom right, EEM#2) longer excitation wavelength (EX WL) range, which are varied from 250–345 nm, 345–450 nm and respectively. The cross symbols (orange-colored fill) in both emission maps indicate the excitation-emission maximum (*λ*_ex_/*λ*_em_ (max)) of the fluorescent components in Ligno-Pulp, and the values are shown (orange-colored rectangular bar) below the corresponding emission maps. (**b**) Representative structure of (left) lignin-carbohydrate complex and (right) softwood lignin, which are marked as no. 1 and no. 2 in the emission maps (Fig. 2a). (**e**) Fluorescence “fingerprint” of lignocellulosic materials and carbohydrates (Fig. 2a, Supplementary Figure S1a,b,c,d,e,f,g), enlisting the *λ*_ex_/*λ*_em_ (max) values, which are indicated as cross symbols on the colored rectangular strip. The light blue and red colored rectangular boxes accentuate the difference between lignin and lignin-free polysaccharides.

Since unbleached pulp fibre (Ligno-Pulp) is composed of lignin, hemicellulose, and cellulose, at first an in-depth EEM characterization of Ligno-Pulp (Fig. 2a) was conducted under two different ranges (250–345 nm and 345–450 nm) of the excitation wavelength (*λ*_ex_) spanning from ultraviolet (UV) to visible light. To ascertain the origin of the excitation and emission maximum concerning the structure of lignin (Fig. 2b), relevant lignocellulosic and carbohydrate reference materials were investigated under similar experimental conditions (Fig. 2c, c*. f.*, Supplementary Figure S1a,b,c,d,e,f,g).

**Figure 3:**
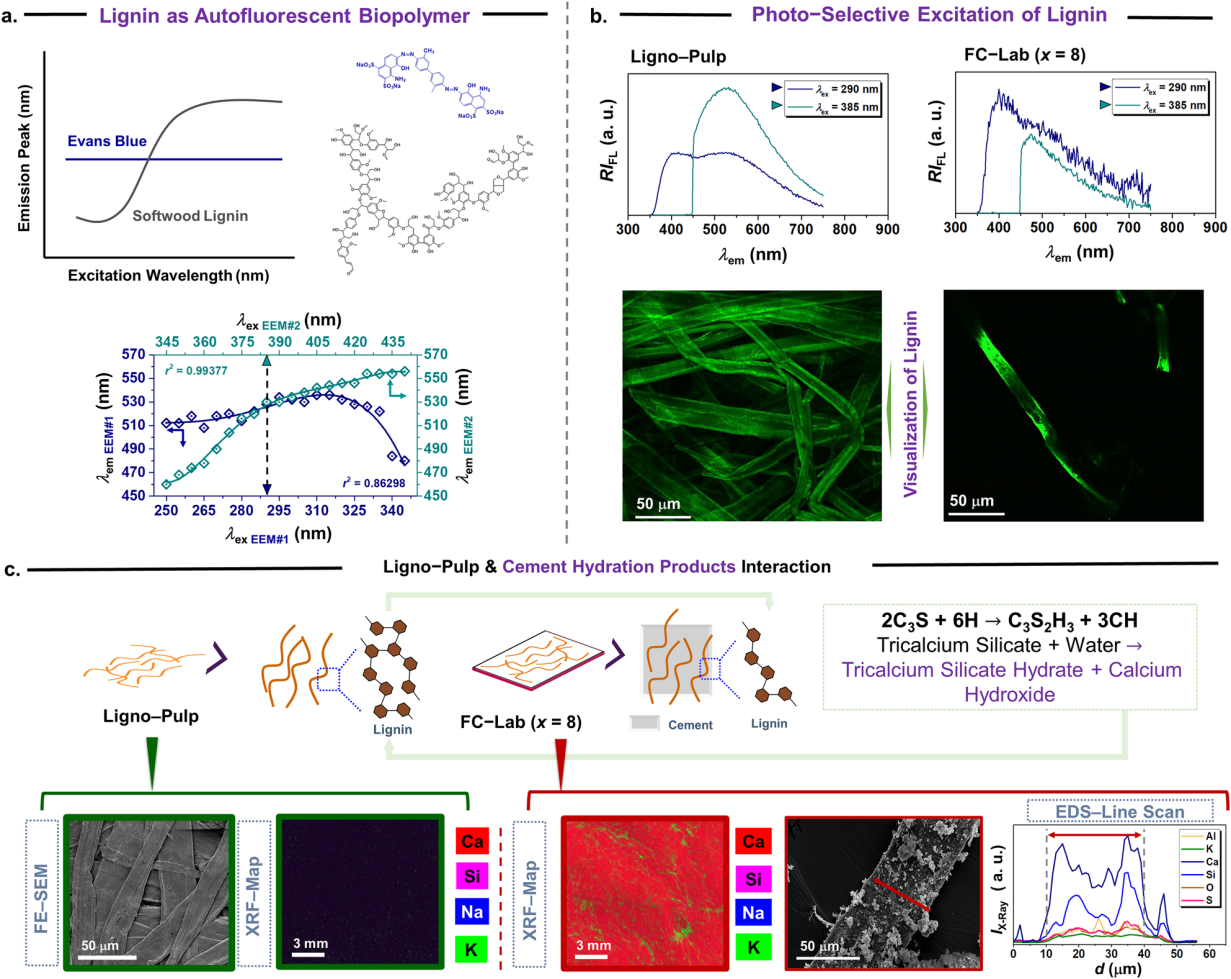
Photo-selective excitation and chemical interaction of lignin as the autofluorescent biopolymer in fibre cement. (**a**) (top) Lignin as autofluorescent biopolymer and (bottom) emission wavelength-excitation wavelength correlation plot of Ligno-Pulp based on the emission maps shown in Fig. 2a. The left Y-axis corresponded to the emission wavelength of EEM#1, whereas the right Y-axis corresponded to the emission wavelength of EEM#2 pertinent to lignin structure (see, Fig. 2b, no. 2) as shown in Fig. 2b. (**b**) Steady-state fluorescence (emission) spectrums of (**b**–**c**) Ligno-Pulp and fibre cement (FC-Lab (*x* = 8)) under 290 and 385 nm (indicated in the bottom and top X-axis of the correlation plot in Fig. 3a). Curves in (bottom) Fig. 3a fitted with a 5th-order polynomial equation. (**c**) Schematic presentation of the chemical interaction between lignin (as a component of Ligno-Pulp) and cement hydration products. The reaction shown in the rectangular box (right-side) refers to the hydration reaction of tricalcium silicate (C3S) with water (H) as a component of cement), which produces tricalcium silicate hydrate (C3H2S3), and calcium hydroxide (CH). Microscopic (e.g., field emission (FE) SEM, and XRF), and spectroscopic (EDS elemental line scan) visualization of the fibre-cement interface. Each XRF-map micrograph shows the distribution of Ca, Si, Na, and K (S and Fe not shown for clarity). The representative SEM micrograph of FC-Lab (*x* = 8) and the corresponding EDX elemental line scan across the width of a pulp fibre (indicated by the mahogany brown colored double-sided arrow. Note that carbon (background) and magnesium X-ray signals were omitted from the line scan results for clarity.

The Fig. 2 illustrates the fluorescence “fingerprint” characteristics of Ligno-Pulp. In Fig. 2a (EEM#1), the bimodal emission profile of Ligno-Pulp was apparent exhibiting two broad (width > 200 nm) peaks at 420 nm (peak#1) and 536 nm (peak#2) while it was monomodal in EEM#2 (Fig. 2b) — both emission maps displayed *λ*_ex_/*λ*_em_ (max) of 325/420 and 410/536, respectively. In addition to the observed excitation and emission maximums, there was a conspicuous (continuous) shift in the emission peak (vide infra, Fig. 3a) of Ligno-Pulp as excitation wavelength was increased from 345 to 440 nm — a classic example of excitation energy transfer, a phenomenon which takes place after the formation of an electronically excited state in a conjugated polymer^35^. Note that the current observation is akin to the findings from Jeffers et. al.^36^, who observed that under 337 nm N_2_-laser excitation, the steady-state fluorescence (emission) spectrum of Ligno-Pulp was also bimodal and the integrated (*λ*_1_ to *λ*_2_) emission intensity correlated with the Kappa number (*K*) for southern pine and mixed hardwoods derived pulp fibre. Also, based on the literature survey, (Kraft) Ligno-Pulp fluorescence “*fingerprint*” was distinctive as opposed to dehydrogenative polymers (DHPs; model lignin)^37^, hardwood lignin^37^, and unbleached mechanical pulp^38^.

Now, such a dramatic change in spectral feature (*I*_em_ and peak *λ*_em_) for Ligno-Pulp could be attributed to the complex structural architecture of lignin, which is intertwined with hemicellulose and cellulose (Fig. 2b). Now, residual lignin structure in the Ligno-Pulp (softwood-derived) is complicated as shown in Fig. 2b (also, see Supplementary Figure S2), which not only bears native lignin structures, like, non-condensed guaiacylglycerol-*β*-guaiacyl ether (*β*-O–4), phenylcoumarane (*β*-5), pinoresinol (*β-β*), but also Kraft pulping process induces the formation of alkali resistant chromophores (Supplementary Figure S2). To name a few, structures like benzyl–alkyl ether (*α*-*O*–alkyl/*β*-O–4) unit, which corresponds to lignin–carbohydrate complex (LCC, Fig. 2b), and stilbene (aryl–vinyl moiety, Supplementary Figure S2) are predominant chromophoric structure in Ligno-Pulp^18,19^. Thus, the change in the spectral profile with EX WL was owing to the presence of these fluorophore and chromophore groups in the residual lignin^39^.

Now, to ascertain the chemical speciation pertinent to *λ*_ex_/*λ*_em_ (max) of 325/420 and 410/536 for Ligno-Pulp, we investigated a wide range of relevant lignocellulosic materials—there are two distinct sets of *λ*_ex_/*λ*_em_ (max) for lignocellulosic materials with and without lignin, which delineate the impact of lignin on pulp fibre fluorescence. For instance, lignin-free cellulosic materials (including hemicellulose) exhibit emission maximum under UV excitation, e.g.,∝–Cellulose displayed *λ*_ex_/*λ*_em_ (max) of 325/440 (Fig. 2c, Supplementary Figure S1a)^39^. On the contrary, lignocellulosic materials exhibits emission maximum under both UV and visible excitation, e.g., Ligno-CNF exhibited *λ*_ex_/*λ*_em_ (max) of 325/424 and 410/524 (Fig. 2c, Supplementary Figure S1c,d).

Overall, EEM (emission map) characterization revealed distinctive *λ*_ex_/*λ*_em_ (max) of lignin in unbleached pulp fibre, and the emission characteristics, such as, *I*_em_ was dependent on the chosen *λ*_ex_, which can serve as the reference to track lignin degradation in fibre cement. Also, as an additional benefit, the combination of high sensitivity and “fingerprint” feature of EEM can enable the detection of lignin impurity in polysaccharides, i.e., xylan. However, since the emission profile is intrinsically broad for unbleached pulp fibre, it is crucial to find a specific excitation wavelength range (vide infra) for lignin so to minimize the issue of spectral overlap between lignin, hemicellulose, and cellulose.

### Photo-selective excitation of lignin in lignocellulosic materials and fibre cement

In this section, we introduce the concept of ‘photo-selective excitation’ based on the emission map (see, Fig. 2a) analysis of Ligno-Pulp, which could be useful in qualitative characterization and site-selective visualization of autofluorescent lignin in composite material, such as fibre cement. Herein, we have proposed a facile and systematic way to elicit the specific excitation wavelength (Fig. 3a) by analyzing the emission peaks from the emission maps as shown in Fig. 2b (EEM#1; *λ*_ex_: 250–345 nm, and EM#2; *λ*_ex_ range: 345–440 nm). It is important to note that such a concept is quite common in fluorescence anisotropy (polarized light), and total internal reflection fluorescence (TIRF) microscopy (angular illumination of light) — effective in understanding size and shape of proteins, and microtubule (microscopic hollow tubes made of the proteins α- and *β*-tubulin) behavior in live mammalian cells.

The Fig. 3 illustrates the extraction process of selective excitation wavelength from the EEM-based emission wavelength-excitation wavelength correlation plot and its implication on steady-state fluorometric detection and visualization of lignin degradation in cement composite (herein, fibre cement to be specific). First, it is important to highlight the key difference between a conventional synthetic dye (i.e., Evans Blue) and a non-conventional lignin-based autofluorescent biopolymer. As graphically shown in (top left) Fig. 3a, in the case of Evans blue dye (mixed with calcofluor white), the position of the emission peak remains constant (see, Supplementary Figure S3) whereas it is not the case for lignin (as a component in Ligno-Pulp). The structural complexity (intrinsic and engineered) of lignin entails several (i.e., > 3) fluorophore groups, which impart the transfer of excitation energies resulting in a continuous red shift with excitation wavelengths (345–450 nm). Therefore, the constructed correlation plot displaying a non-linear trend (Fig. 3a), and interestingly the two curves intersected at the emission wavelength of 530 nm (note: 536 nm is the emission maximum), corresponding to 290 nm (UVB light) and 385 nm (violet light) excitation wavelengths, respectively. Note that the G-lignin-based model lignin compound also showed selectivity towards violet light^37^. Indeed, 290/530 and 385/530 do not correspond to *λ*_ex_/*λ*_em_ (max) of lignin (as a component in Ligno-Pulp) but the observed selective excitation wavelengths provide an opportunity to narrow down the range of excitation wavelength, that is 385–410 nm for lignin.

Next, as model examples, we tested lignin autofluorescence in Ligno-Pulp and a fibre cement (FC-lab (*x* = 8)). As shown in Fig. 3b, upon overlaying the emission spectrums, the emission intensity (above 500 nm) was twice as high at 385 nm than at 290 nm EX WL indicating the “photo-selectivity” of residual lignin in Ligno-Pulp. On the other hand, in the prototype fibre cement (Fig. 3b), the bimodal emission profile of lignin was not apparent and emission intensity (above 500 nm) ~ 20% lower at 290 nm versus 385 nm excitation wavelength — an indication of lignin degradation/dissolution. Now, one can argue about the contribution from the cement as a matrix; cement particles (also C–S–H) fluoresce under near infra-red (NIR) excitation as reported by Weissman et. al.^40,41^. They observed that under excitation between 500–1000 nm, Portland cement (independent of cement hydration state, aggregates, and mechanical strain) exhibited emission peak at 1140 nm (width ~ 30 nm). It was hypothesized that surface texturing (during clinker formation)^40^ and/or nano structuration of silica contributed to the strong NIR emission^42^. Furthermore, using 2–P microscopy (laser excitation: 400 nm), lignin autofluorescence was visualized for both Ligno-Pulp and fibre cement, but it was not possible to discern whether lignin is degraded in the cement composite or not. Further studies are warranted in this regard.

In the case of fibre cement, as a physical component, cement could reduce the overall intensity of lignin since pulp fibre is employed at particular composition (i.e., 8 wt.%). However, it must not interfere emission spectrum feature (e.g., emission maximum) of the lignin unless there is a chemical interaction. Cement, when it contacts water produces cement hydration products, namely, calcium silicate hydrate (C–S–H) and calcium hydroxide (CH) as shown in the Fig. 3c graphic (top right), which will physically adhere to the surface of pulp fibre (see, SEM micrographs in Fig. 3c). Thus, the creation of fibre-cement interface affords the bonding between the reinforcing fibre the brittle matrix, essential to the mechanical strength development of the composite. Henceforth, to provide evidence about the formation of the cementitious components via a combination of three x-rays (micro) spectroscopic techniques, which were x-ray diffraction (XRD) (Supplementary Figure S4), x-ray fluorescence (XRF) (Fig. 3c), and energy dispersive x-ray (EDX) spectroscopy (Fig. 3c). At first, XRD confirmed the formation of CH, and C–S–H in fibre cement (pulp fibre content, *x* = 8, 16, and 32) alongside calcium carbonate (ettringite) and other cementitious species (Supplementary Figure S4). Then, the XRF map of FC-Lab (*x* = 8) surface depicted the formation of a calcium-rich surface (spot size ~ 25 μm) with the colocalization of silicon, potassium, and sodium, which were absent in Ligno-Pulp (Fig. 3c). Since crystallization of CH triggers the alkaline attack in fibre capillaries, hence, energy dispersive x-ray (EDX) spectroscopy (penetration depth ~ 10 nm) was employed on of isolated pulp fibre. Upon EDX-line scan (Fig. 3c, bottom right), the intensity of the emitted x-ray intensity from calcium (*K*_*∝*_ = 3.69 eV) and silicon (*K*_*∝*_ = 1.739 eV) were higher than other elements (e.g., Al (*K*_*∝*_ = 1.486)) indicating possible colocalization of CH and C–S–H on pulp fibre surface, a classic scenario of “fibre lumen mineralization^42^”.

Overall, monitoring emission intensity under from 385 to 410 nm excitation wavelengths affords the decoupling of fluorescence of lignin from its polysaccharide counterparts, e.g., cellulose — degradation of lignin in fibre cement by the alkaline component (CH) of cement hydration products is possible to probe from 385 to 410 nm excitation wavelengths. To be specific, 385 nm (violet light) excitation wavelength is suitable to monitor (qualitatively) lignin degradation in composite materials or in other matrix environments where lignin is exposed to strong alkalis or acids. However, it lacks the quantitative aspect of lignin degradation, which could be alleviated via in-depth EEM characterization of fibre cement.

### EEM (Emission map) characterization of fibre cement and eem-based monitoring of lignin degradation

In this section, we have characterized fibre cement using EEM (*λ*_ex_: 345–450 nm) and proposed a quantitative method to monitor lignin degradation. To achieve this, content (*x*) of pulp fibre was varied (*x* = 8, 16, and 32) in the fabricated cement composite. Note that since cement matrix can reduce the overall fluorescence (emission) intensity of lignin (autofluorescent tag) without any chemical effect, we compared each fibre cement EEM against a dry mixture (DM) of cement and pulp fibre (see, experimental for details). In such a way, we can calculate “true” lignin degradation, which is down to the chemical effect of cement hydration products (Fig. 3c) — absent in DM since no water was added.

**Figure 4:**
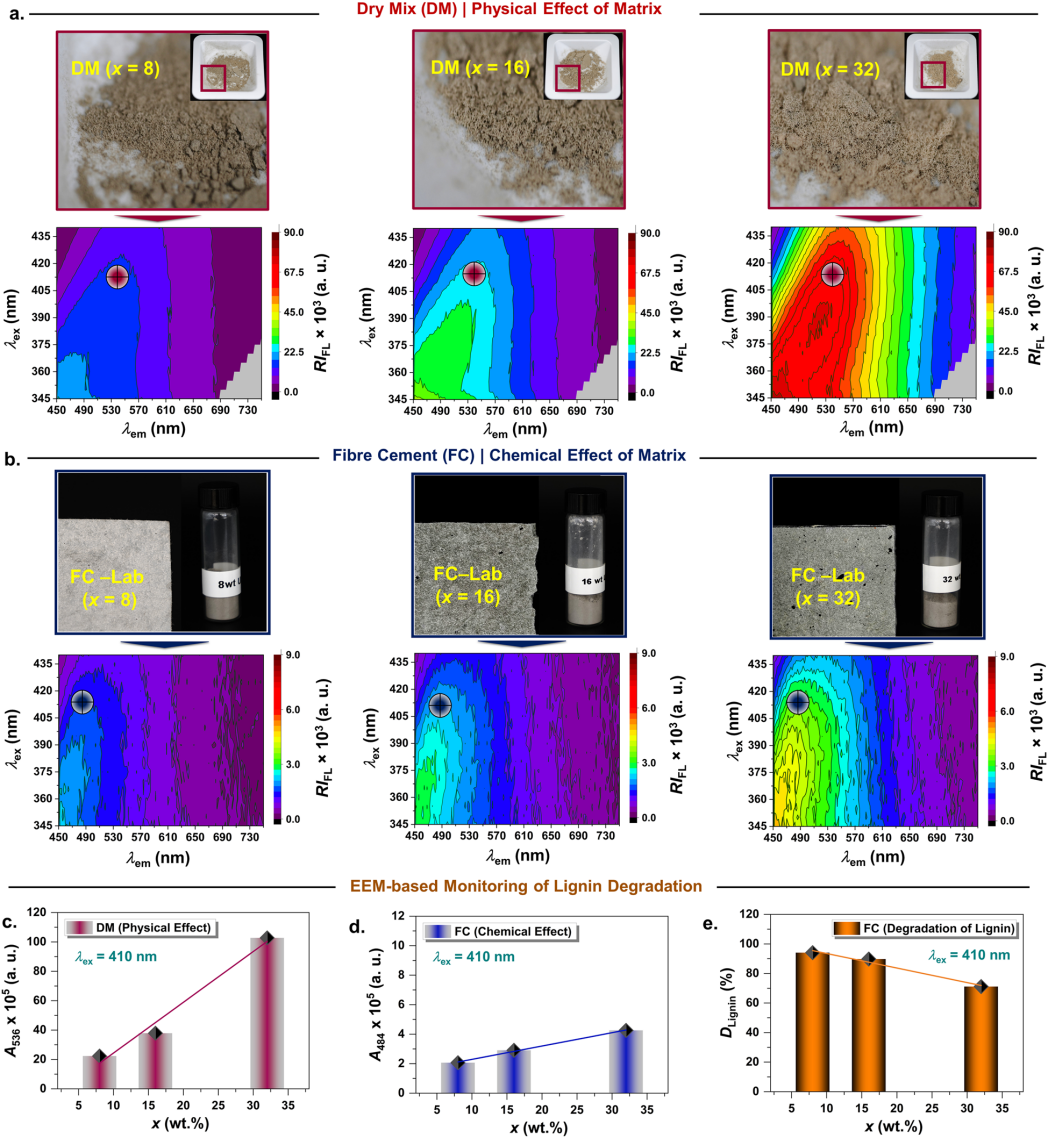
EEM (emission map) characterization of fibre cement and EEM-based Monitoring of Lignin Degradation as a Function of Pulp Fibre Content. EEM of (**a**) dry mix (DM) of unbleached pulp fibre (Ligno-Pulp) and cement, (**b**) fibre cement (FC). The digital photographs correspond to each EEM shown in (**a**) and (**b**). Integrated area (*A*) intensity of the emission spectrum corresponded to lignin in (**c**) DM, and (**d**) fibre cement at 410 nm excitation wavelength. The emission peak corresponding to the area calculation is indicated by the cross-symbols on the emission maps in (**a**) and (**b**). (**e**) Calculated degree of lignin degradation (*D*_Lignin_) based on the integrated area (*A*) intensity accounting for the chemical effect of cement hydration products. Note that *D*_Lignin_ corresponds to the difference between integrated area (*A*) intensity between Ligno-Pulp (Fig. 2b, EEM#2) and fibre cement (corrected by the physical effect of cement matrix), see, method section for details.

The Fig. 4 illustrates the EEM of pulp fibre lignin in two different microenvironments mixed with cementitious materials as a function of pulp fibre content. As shown in Fig. 4a, the fluorescence “fingerprint” of lignin for DM samples is similar (meager change in *λ*_ex_/*λ*_em_ (max)) to the one observed for Ligno-Pulp (Fig. 2a, EEM#2) irrespective of the pulp fibre content. But the emission intensity was an order magnitude (10^1^) lower — the physical effect of the cement matrix. Interestingly, lignin fluorescence in (dry) cement exhibited high sensitivity even at lower content (i.e., DM (*x* = 8)). Next, as shown in Fig. 4b, the emission maps of fibre cement (FC) were distinctive (irrespective of the pulp fibre content) — the absence of emission maximum with emission tailing off from > 500 nm and emission intensity was reduced 10 times compared to the DM samples — chemical effect of the cement matrix. Note that the fibre cement samples were cured for 7 d and the emission map results confirm that even at the early curing stage a rapid alkaline attack on lignin has occurred, which we believe will slow down as fibre cement hardens with time (curing time for load-bearing solid is > 28 d). Based on the earlier studies^43^, it has been evident that cement hydration products (i.e., calcium hydroxide, CH) first target lignin before attacking the hemicellulose, and cellulose; then disrupt the link between the fibre cell walls upon dissolving the lignin. The phenomenon is known as “fibre lumen mineralization”^34^, which is primarily responsible for causing the embrittlement of the composite during wet-dry cycle tests (e.g., sisal fibre reinforced cement).

To this end, for quantitative lignin degradation analysis, we set two criteria, first is the selection of a fixed excitation wavelength, that is, 410 nm (*λ*_ex_ (max) for Ligno-Pulp), and the second is the use of an integrated area intensity rather than peak intensity. As the area under the emission curve (at photo-selective excitation) represents solely the emission (fluorescence) from lignin, via this method, we can estimate the overall lignin degradation in fibre cement. Interestingly, the integrated area (*A*) intensity of both DM (*A*_536_) and FC samples (*A*_484_) displayed a linear trend as a function of pulp fibre content (*x*) demonstrating the excellent sensitivity of the method without any significant inner-filter effect (synonymous in fluorometry of concentrated solution)^44^. Now, degradation of lignin (*D*_Lignin_) means how much lignin autofluorescence (in terms of integrated area (*A*) intensity) was reduced in fibre cement. (See, method section for calculation details). As seen in Fig. 4c (right), for fibre cement with 8 wt.% of reinforcement (Ligno-Pulp), *D*_Lignin_ (%) was ca. 94%, while it was ca. 71% when reinforcement was 32 wt.%. Thus, it was conspicuous that the “shielding” effect of lignin is weak, especially at low Ligno-Pulp content (FC-Lab (*x* = 8)). However, one must be wary that at high pulp fibre content (*x* = 32) mechanical strength could be compromised simply owing to the absence of suitable filler (reduces void) and superplasticizer (improves fibre dispersion). For instance, the modulus of rupture (MOR) of FC-Lab (*x* = 32) was reduced by ~ 60% when compared with FC-Lab (*x* = 8) (Supplementary Figure S5). Note that MOR is a bulk property whereas fluorometry provides molecular-level information about individual fibre component, i.e., lignin and its fate upon interacting with the cement hydration products.

In general, there are a few caveats of the proposed method and caution is pertinent as *D*_Lignin_ (%) delineates the overall decrease in lignin content where the intensity drop could stem not only from the breakdown of the polymeric backbone of lignin but also from the chromophore groups. We note that one of the chromophore groups which could hinder the fluorometric observation is 4-deoxy-4-hexenuronic acid (HexA), usually formed from the partial conversion of 4-O-methyl glucuronic acid groups in xylans during Kraft pulping, often found in with mid-range Kappa number (*K* > 25, lignin content > 5 wt.%)^45–47^. We anticipate that in Ligno-Pulp with low-range *K* (value of 23.4), HexA content would be a minor component and since one of the cement hydration products, i.e., calcium hydroxide (CH) forms a high-alkaline ([OH]^−^: 700 mmol/L versus 275 mmol/L in Kraft process) buffer solution^48^ — CH will certainly attack any labile unsaturated unit in the residual lignin structure of Ligno-Pulp, which is detrimental to the stability of natural fibre in fibre cement^49^. Wei and Meyer ^47^ proposed a degradation mechanism of natural fibre by CH mineralization on fibre lumen, which leads to the loss of mechanical strength pertinent to the pulp fibre (reinforcement).

To corroborate the findings for the fibre cement fabricated at the lab, the recorded EEMs (Supplementary Figure S6a,b,c,d) of a commercial fibre cement (FC-Commercial) exhibiting similar EEM characteristics (i.e., the reduced intensity at excitation > 345 nm, Supplementary Figure S6b, d) to that of FC-Lab (Fig. 4b) — fluorescence “*fingerprint*” from EEM can be utilized to investigate chemical stability of lignocellulosic fibre in a cementitious composite. Interestingly, FC-Commercial was more emissive (above 500 nm, *c. f.* Supplementary Figure S6d) than lab-made fibre cement (Fig. 4b), which is a testament to the stronger “shielding” effect of lignin in the former than in the latter. Interestingly, the normalized emission spectrums of lab-made and commercial fibre cement were identical at 410 nm excitation wavelength (Supplementary Figure ﻿S7). It is worth mentioning that the commercial high-density fibre cement does not contain fibre reinforcement > 8 wt.%. Thus, we hypothesize that it could be either lignocellulosic fibre had higher Kappa number (*K* > 23.4) than the Ligno-Pulp (*K* = 23.4) used in this study or silica/clay-based additives were employed during the fabrication process. We note that metakaolin (calcined clay) rendered improvement of fibre stability in fibre cement owing to its high content (50 wt.%) of reactive silica^42^.

Overall, EEM-based monitoring of lignin degradation provides a quantitative method for understanding of the lignin-cement hydration products interaction spectroscopically — a high value of *D*_Lignin_ (%) could be a “pre-warning” when the longevity of natural (lignocellulosic) fibre in the cementitious matrix is of paramount importance. ﻿Keep in mind that variability of lignin composition in the raw materials, i.e., Ligno-Pulp must be checked (see, Supplementary Figure S8) prior to the in-depth EEM investigations owing to its high sensitivity. Also, lignin degradation in both lab-made and commercial fibre cement possibly occurred via a specific mechanism (i.e., alkaline hydrolysis) supporting the observation from Wei and Meyer^47^. Note that we have emphasized on shorter curing period to establish the fluorometry-based technological platform. Temporal lignin degradation over longer curing time will be the subject of the next manuscript, which is currently underway in our research group. However, EEM cannot ascertain the breakdown of lignin’s conjugated polymeric structure, which require the implementation of a more selective multidimensional fluorometric technique, such as TSFS (vide infra).

### TSFS (Synchronous map) characterization of lignocellulosic materials and fibre cement

Although EEM is powerful in discerning different autofluorescent materials (e.g., lignin and α-cellulose), it has limitations in terms of selectivity towards conjugated polymeric structures, such as lignin. Thus, in this section, we present the total synchronous fluorescence spectroscopy (TSFS) or synchronous map-based characterization of unbleached pulp fibre (Ligno-pulp), lignocellulosic nanofibre (Ligno-CNF), lignin model compound, that is, guaiacylglycerol-β-guaiacyl ether (*β*-O–4 units) has been employed to ascertain about the breakdown of lignin’s macromolecular structure.

**Figure 5:**
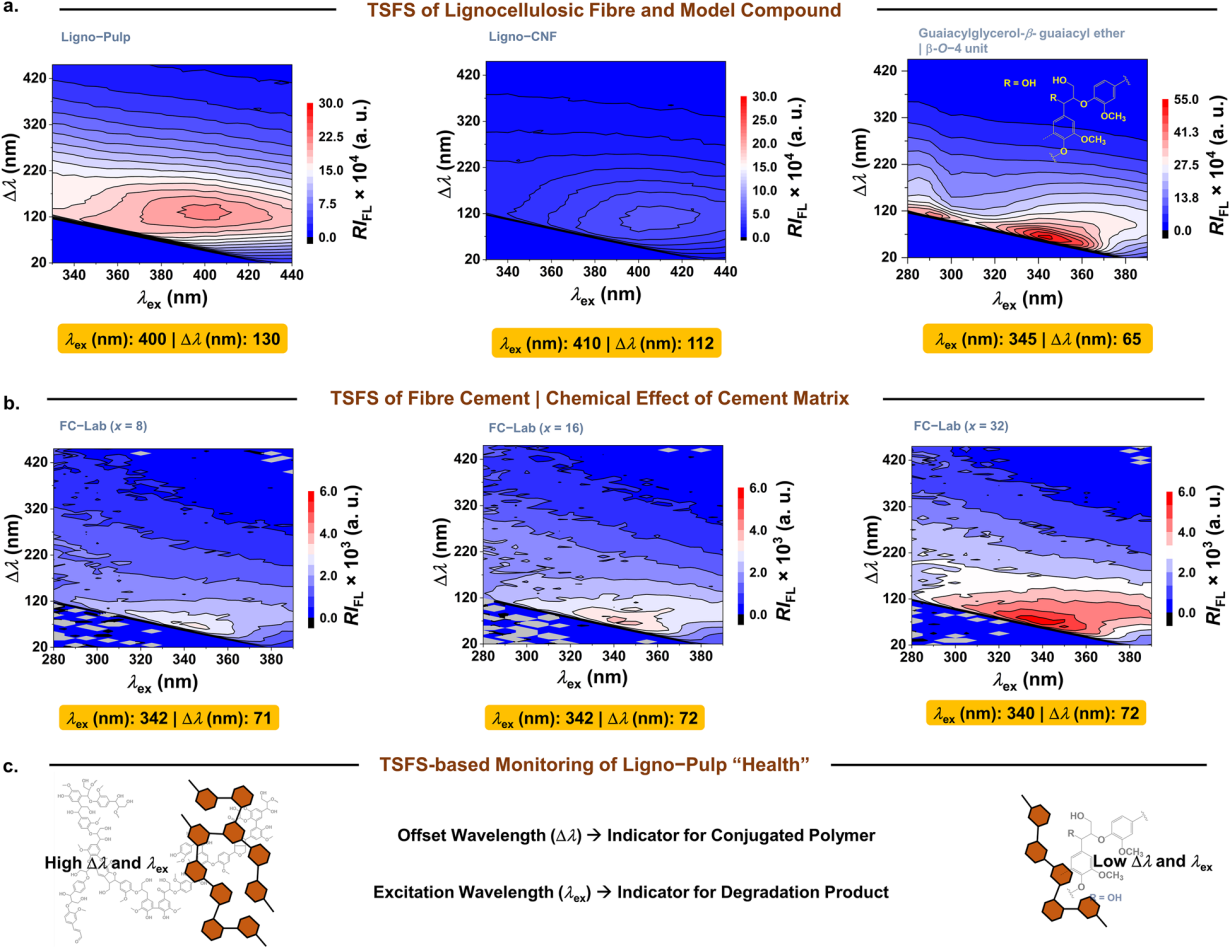
TSFS (synchronous map) characterization of lignocellulosic materials and fibre cement. Synchronous map of (**a**) Lignocellulosic materials and lignin model compound (**b**) fibre cement as a function of pulp fibre content. The range of excitation wavelength in (**a**) and in (**b**) were 350–450 nm and 250–390 nm, respectively. Both maximum excitation wavelength (*λ*_ex_) and offset wavelength (∆*λ*) are shown (orange colored rectangular box) below the corresponding to the synchronous map in (**a**) and (**b**). Maximum emission wavelength (nm), *λ*_em  _= ∆*λ* + * λ*_ex_ (**c**) Schematic presentation of the TSFS-based monitoring of pulp-fibre “health” in terms of excitation wavelength (*λ*_ex_) and offset wavelength (∆*λ*). Specific long-wavelength-pass filters (LP400) was employed during the acquisition of the synchronous map.

Figure 5 illustrates the TSFS-based characterization of lignocellulosic materials and fibre cement and it provides maximum excitation wavelength and offset wavelength as monitoring parameters to probe the degradation of conjugated polymeric structure of Ligno-pulp pulp fibre “health” in the cement matrix. For example, as shown in Fig. 5a, Ligno-Pulp and Ligno-CNF both exhibited maximum excitation wavelength ca. > 400 nm and offset wavelength ca. > 110 nm whereas, guaiacylglycerol-β-guaiacyl ether (lignin model compound) displayed at 345 nm, and 65 nm, respectively. Intriguingly, despite the difference (~ 5 wt.% and 13 wt.%, respectively) in lignin content for Ligno-Pulp and Ligno-CNF, *λ*_ex_/*λ*_em_ (max) from TSFS was similar — effective conjugation length (ECL) could be close owing to the low molecular weight of residual lignin in these lignocellulosic materials^49^.

Note that light absorption of organic compounds is affected by the conjugation and usually with increased conjugation (i.e., the addition of conjugated double bond), the absorption (excitation) spectrum will be shifted to a longer wavelength (bathochromic shift). To this end, we note that literature on solid-state TSFS, especially on degradation studies of conjugated polymers is scarce. However, Donaldson^50^ observed, a “bathochromic shift” (longer wavelength) in emission > 500 nm (red and far-red) in compression wood owing to the increase of conjugation length of lignin fluorophores per free-electron molecular theory (FEMO). Now, based on FEMO, spectral change (i.e., band shift) depends on the length of the conjugated chains and it does not account for the unconjugated fluorophores. Thus, owing to the higher selectivity of TSFS (versus EEM), the breakdown of the conjugated structure of lignin must result in a “hypsochromic shift” (shorter wavelength) in the observed *λ*_ex_ and, ∆*λ*.

In our observation, as shown in Fig. 5b, in the case of fibre cement, both of these parameters displayed “hypsochromic shift” (i.e., *λ*_ex_ wavelength of 342 nm, and ∆*λ* 71 nm for ﻿8 wt.% of Ligno-Pulp). One must conder that in solid state diffusion of the degraded products is limited as opposed to the diffusion in a solvated environment (solution). For example, Bartolomei et. al.^16^, reported that lignin oligomers (4–6 aromatic rings) exhibited *λ*_em_ (max) ~ 350 nm under only 20 nm offset wavelength (∆*λ*) and the emission wavelength of conjugated lignin showed “hypsochromic shift” upon catalytic (Pt/C) depolymerization in solution. Therefore, it is more challenging to identify the chemical speciation of the degraded lignin products in solid state. Remarkably, the synchronous map of fibre cement sample (irrespective of pulp fibre content) exhibited emission maximums under offset and excitation wavelengths similar to that of guaiacylglycerol-β-guaiacyl ether (Fig. 4a, top right) — confirming the ﻿structural degradation of polymeric lignin. The change in high ﻿offset wavelength (i.e., from 130 nm to ~﻿ 72 nm) for Ligno-Pulp indicates disruption in the conjugated framework of lignin while change in high excitation wavelength (i.e., from 400 nm ~﻿ 342 nm) indicates formation of non-polymeric degradation product (as illustrated in Fig. 5c) in fibre cement.

The proportion of *β*-O–4, and *β*-5 units in the residual lignin structure decreases during Kraft pulping (alkaline delignification). Concurrently, degraded products (secoisolariciresinol type (*β-β*), dihydroconiferyl alcohol moieties) from *β*-O–4 unit, and conjugated carbonyl groups at terminal side chains are dominant in (Kraft) Ligno-Pulp ^18,19^. We speculate that the remainder *β*-5 units and conjugated carbonyl groups are possibly degraded (alkaline hydrolysis) during the initial stage of curing. Although *β*-O–4 and *β*-5 content is low, owing to the high sensitivity of TSFS, the “hypsochromic shift” of the offset and excitation wavelengths were conspicuous and large (~ 60 nm) in fibre cement. On the contrary, alkali resistant *β-β* linked moiety with the accumulation of aryl–vinyl moiety (stilbene) and condensed (stable carbon–carbon bonds) structures, such as (∝-5), biphenyl (5–5), aryl-*O*-aryl (4-O–5) could remain intact in fibre cement, but at this moment, we do not have any additional evidence^18,19^. Thus, like EEM, a synchronous map-based database of lignin model compounds is also warranted to further investigate the applicability of excitation and offset wavelengths as monitoring parameters for in-depth understanding of the degradation mechanism (and products) of lignin in fibre cement, which is beyond the scope of this manuscript.

## Conclusions

In this study, multidimensional fluorometric techniques (EEM and TSFS) enabled rapid and label-free investigation pulp fibre “health” (indicator: lignin) in cement matrix by leveraging autofluorescence of lignin. We discovered that the concept of “*photo-selective excitation*” is crucial in discerning lignin autofluorescence from cellulose and/or LCC. XRF, SEM–EDX, and powder XRD provided the evidence that Ca and Si-rich cementitious components, which mostly CH (calcium hydroxide), and C–S–H were deposited onto the surface of unbleached pulp fibre, of which calcium hydroxide could degrade lignin via alkaline hydrolysis. Based on EEM, we observed a reduction (from ~﻿ 94% to ~﻿ 71%) in the degree of lignin degradation when pulp fibre content was increased from 8 wt.% to 32 wt.%. Furthermore, TSFS (synchronous map), confirmed the breakdown of lignin’s polymeric (conjugated) structure demonstrating “hypsochromic shift” (~ 60 nm) of the offset and excitation wavelengths for fibre cement when compared with pristine pulp fibre. Overall, we believe that the findings of this study will provide an opportunity to utilize multidimensional fluorometry as a platform technology to “fine-tune” the lignocellulosic fibre-cement interface for the germination of low-carbon natural fibre reinforced composites.

## Materials and methods

### Materials

Ordinary Portland cement (OPC) was from Lafarge (Vancouver, Canada). Unbleached Kraft pulp sheet/Ligno-Pulp (1 mm thickness) with Kappa number (*K*) of 23.3 (trade name: UB–Cement™) and 21.6 (trade name: UB–Elite™), Bleached-Pulp (bleached NBSK) and waste unbleached pulp fibre (W-Ligno-Pulp) were from Canfor (Prince George pulp mill, Canada). Physical properties of the unbleached pulp fibre (Ligno-Pulp) are enlisted in the Supplementary Table S1. Ligno-CNF was prepared per the report from Imani et al.^49^ Reverse osmosis (RO) water was employed for soaking the pulp sheet (before refining) and for preparing the fibre cement slurry. ∝-Cellulose, guaiacylglycerol-beta-guaiacyl ether, and Ligno-Xylan (4-*O*-Methyl-D-glucurono-D-xylan), Evans blue (dye content ≥ 75%) were from Sigma-Aldrich (Canada).

### Fabrication of the fibre cement

The lab-scale procedure of fabricating the fibre cement was inspired by the commercial (semi-continuous mode) Hatschek process and to minimize the sample wastage and to have a good representation while transferring the fibre cement slurry to the mold (nickel plated stainless-steel, model: Elegance Silver 82,533) for casting, a required amount of OPC, refined (PFI mill, 2500 rpm) Ligno-Pulp and RO water was mixed in a tabletop kitchen mixer (Techwood electric stand mixer, model: TWSC-263, 6-speed mode) with a bowl (stainless-steel, capacity: 6.0 QT) and a flat beater (aluminum alloy) as the impeller. After a week, fibre cement naturally comes off from the mold without the aid of a releasing agent. After that, we kept it inside of a reclosable plastic bag (details in Supplementary file).

### Sample preparation for fluorometric characterization

The interface of fabricated fibre cement (thickness: 8 mm) is challenging to investigate as is via spectroscopic and microscopic techniques. As such, we cut the fibre cement per to the ASTM C1185 method into three rectangular bars, then broke them by a hammer into small pieces before the mild pulverization with the blender (model: Black + Decker, 350 W, mode: ice crushing) for only 2 min (30 s pulse) to separate the pulp fibre from the cement matrix. To minimize the scattering artifacts, packing of the materials (particles and/or fibres) is crucial, hence, the specimen was sieved (Cole-Parmer 3"-Diameter Sieve) with a size of 150 mm (100 mesh screen, U.S. mesh size). Sample (e.g., fibre cement) mass was ~ 60 mg during fluorometric characterization. Note that the preparation of the powder (and micronized fibre) samples was the same except for the pulp sheet (1 mm thickness). To compare with the Ligno-Pulp, a solution (concentration: 0.5 g/L) of Evans of dye was also prepared.

### Fluorescence spectroscopy

A fluorometer (Edinburgh instruments, model: FLS1000) using a 450 W xenon arc lamp as the light source (range: 230–1000 nm) was employed for the fluorescence spectroscopy. A specifically designed sample holder for solid state materials positioned at 45° relative to the photomultiplier tube (PMT) detector (model: PMT-980, spectral range: 185–980 nm) affording the collection of the emission signal from the surface since Ligno-Pulp and cement are dense and opaque (see, Supplementary for details).

### Scanning electron microscopy and energy dispersive X-ray spectroscopy

A scanning electron microscope (SEM, model: FEI™ Helios NanoLab 650) with an EDAX TEAM™ Pegasus system (AMETEK, Inc.) was employed by varying the acceleration voltage from 5 and 15 kv. Elemental line scan results (X-ray intensity versus distance) processed by using the EDAX's TEAM™ software for eight elements (C, O, Mg, Al, Si, S, K, and Ca) with the field size of 150 × 120 microns at 0.2 s dwell time (see, Supplementary for details).

### Degree of lignin degradation

The degree of lignin degradation (*D*_Lignin_) is the reduction (%) of integrated area (*A*) intensity of the emission spectrum at 410 nm excitation wavelength nm for fibre cement, if compared with unbleached pulp fibre (Ligno-Pulp). Thus, based on this definition, the value of *A*_536_ nm was chosen as the reference (see, Fig. 2b, EEM#2), which is presented by Eq. (1).

1 $${D_{Lignin}}\left( \% \right) = {A_{536}}\left( {\text{Ligno-Pulp}} \right) - A\left( {\text{F-corr.}} \right)/{A_{536}}\left( {\text{Ligno-Pulp}} \right) \times 100$$

In Eq. (1), *A* (FC-corr.) refers to the reduced integrated area (*A*) intensity of lignin in fibre cement. To calculate it, physical mixtures of pulp fibre and cement, that is labeled as Dry Mix (DM) were prepared with identical pulp fibre content to that of fibre cement (FC). And the difference *A*_536_ (DM)–*A*_484_ (FC) corresponds to actual lignin degradation (denoted as *A* (FC-corr.)) owing to the “chemical effect” of cement hydration products (i.e., calcium hydroxide). Without this correction (corr.), reduction in integrated area (*A*) for fibre cement can be ascribed to the (physical) matrix effect as well.

## Supplementary Information

Supplementary Information.

## Data Availability

The datasets used and/or analysed during the current study available from the corresponding author on reasonable request.
