# Supplementary material for: Unraveling lignin degradation in fibre cement via multidimensional fluorometry
Source: Sci Rep. 2023 May 24;13:8385. doi: 10.1038/s41598-023-35560-3 (PMC10209091; doi:10.1038/s41598-023-35560-3)
Supplement: Supplementary file 1 — Supplementary Information. [file 41598_2023_35560_MOESM1_ESM.docx]

**Electronic Supporting Information**

**Unraveling Lignin Degradation in Fibre Cement via Multidimensional Fluorometry**

**Mahfuzul Hoque^1^, Saeid Kamal^2^, Sreenath Raghunath^1^, and E. Johan Foster^1,3*^**

^1^Department of Chemical and Biological Engineering, The University of British Columbia. 2385 East Mall, Pulp and Paper Centre, V6T 1Z4.

^2^Laboratory for Advanced Spectroscopy and Imaging Research (LASIR), Department of Chemistry, The University of British Columbia. 2036 Main Mall, Vancouver, BC Canada V6T 1Z1

^3^Bioproducts Institute, 2385 East Mall Vancouver, BC Canada V6T 1Z4

Correspondence and requests for materials should be addressed to E. J. F (email: [johan.foster@ubc.ca](mailto:johan.foster@ubc.ca))

Email Address: [johan.foster@ubc.ca](mailto:johan.foster@ubc.ca)

**Materials and Methods**

**Fabrication of the Fibre Cement.** Note that the fibre-cement slurry was prepared inside of a fume hood by keeping water to cement ration constant (w/c = 0.3). The sequence of the added raw materials is as follows, refined Ligno-Pulp, OPC, and RO water. It is important to note that we added half of the OPC and water at first, mixed with the refined Ligno-Pulp for three minutes (speed setting: 6). Then, using a polypropylene plastic spoon, we scaped the slurry from the mixer bowl (side and bottom) and stirred for 30 seconds. Finally, we added the rest of the OPC and water, and then mixed for three minutes, scrapped, and stirred like before. As an ultimate step, the slurry was then continuously mixed for six minutes (speed setting: 6) without taking any further stoppage. Thus, the total mixing time was 12 minutes as longer mixing time will result settling of the slurry inside of the mixer bowl. Note that scraping of the slurry mix is of paramount importance pertaining to its highly viscose nature — more so when high proportion (in wt.%) of refined Ligno-Pulp is employed, fibres tend to stick at the bottom. Thus, it is our recommendation that addition of components must be stepwise and take stoppage in between (not all at once) the mixing process.^59^ Besides, it generates less splashing of the cement powders or any supplementary additives (if employed), ensuing a safer working environment for the users at the laboratory space. After finishing the mixing process, we transferred the fibre-cement slurry into the designated mold (no releasing agent was employed) and using an angled spatula (stainless-steel, model: ANAEAT icing spatula) we leveled the top surface was and then we compacted it using a mini-shakers (Fisherbrand^TM^, without the top hatch) for 15 minutes at 250 rpm. Finally, the fibre cement sample was air cured (temperature = 22 °C, relative humidity = 70%) for 28 d.

**Sample Preparation for Fluorometric Characterization.** Ligno-Pulp was cut into U-shaped slice (~ 60 mg) by using a scissor prior to the fluorometric characterization (slice no. #1 – #7, Supplementary **Fig.** **S5**a-b). In the case of dry mix (water-free) of pulp fibre and cement, pulp sheet was micronized using a blender (model: IKA A11 basic). Then, the micronized pulp and cement particles were weighted proportionately (pulp fibre content was varied from 8 wt.% to 32 wt.%) and then hand mixed with a stirring rod. After that, the physical mixture of pulp fibre and cement (denoted as Dry Mix) was sieved with the same sieve used for fibre cement samples (see, main article, methods section).

**Experimental**

**Mechanical Characterization.** The three-point bending mechanical tests were performed using universal testing machine (UTM) (model: Instron 5969) per to the ASTM C 1185. In this case, the sample was air cured for 28 d prior testing and from the stress-strain curves, modulus of rupture was calculated for the lab-made and commercial fibre cement samples (Supplementary **Fig. S5**).

**Fluorescence Spectroscopy.** During the acquisition of EEM and synchronous map (TSFS), long-wavelength-pass filters (LP450, Thorlabs, LP370) were employed to block the specularly reflected EX WL while transmitting the longer wavelength fluorescence. Temperature was ~ 20 °C during all fluorometric measurements. Optimization of the experimental parameters (e. g., bandwidth, dwell time) ensured the minimum effect of scattering and photobleaching by optimizing while by using the Fluoracle^®^ software, raw data, and three-dimensional plots, and EEMs (two-dimensional contour plots) later processed with Origin (OriginPro^®^ 9.0). For the EEM, the step size for the EX WL and EM WL was 5 nm and 2 nm, respectively. Note that to account the any change in emission intensity, which could simply be ascribed to the natural variation of lignin content within the pulp fibre, we recorded emission spectrums at several locations of the pulp with Kappa number (*K*) of 21.6 (by cutting them into slices, labeled as slice #number in **Fig. S8**a-b) and there was about 1.5% variation in fluorescence intensity (**Fig. S8**b).

**X-ray Fluorescence Microscopy.** A micro-XRF (model: Bruker M4 Tornado) was employed for the x-ray fluorescence microscopy in which the x-ray tube operated at 50 kV and 600 μA. A dynamic sample stage was employed for XRF mapping moving the stage at 1.7 mm/s with pixel time of 10ms/pixel; overall collecting 800 k pixel. The recorded elemental XRF map originally corresponding to six elements (Ca, Si, Na, K, S, and Fe) while in the overlay XRF micrographs, only Ca (major), Si (major), Na (minor), and K (minor) shown for clarity.

**Two Photon Microscopy.** A laser scanning microscope (LSM) (model: Zeiss LSM 510 MP) with a femtosecond laser (model: Coherent Chameleon) as the two-photon excitation source was employed for the visualization of lignin autofluorescence in Ligno-Pulp and fibre cement. The EX WL was tunable from 680–980 nm and laser pulse duration were 120 fs.

**Scanning Electron Microscopy and Energy Dispersive X-ray Spectroscopy.** The scanning electron microscope (SEM, model: FEI^TM^ Helios NanoLab 650) with an EDAX TEAM™ Pegasus system (AMETEK, Inc.) was capable of both energy dispersive spectroscopy (EDS) and electron back scatter diffraction (EBSD) based elemental mapping using a forward scattering silicon drift detector (SDD) and Hikari XP camera, respectively. Prior to SEM imaging, all samples (non-conductive) coated with thin layer (usual thickness ~ 5 nm) of Iridium (Ir) using a direct current (DC) magnetron sputter coater (Leica, model: EM MED020) to suppress accumulation of charges when electron beam hits the sample (charging effect). Prior to the coating, we fixed the samples (pulp sheet, powder particles, and micronized fibres) on the SEM sample stub (TED PELLA, Inc, Ø12.7 mm) with 3.2 mm pin using the carbon tape (TED PELLA, Inc, Ø12.0 mm outside diameter (OD)).

**X-ray Diffractometry.** Powder X-Ray diffraction (PXRD) was performed using an X-ray diffractometer (model: Bruker D8-advance) in a reflective geometry, distance between the sample and the detector was constant (Bragg-Brentano configuration). The x-ray generator was operating at 40 kV and 40 A and the fibre cement samples (powdered and sieved) remained flat (standard Bruker sample holder) throughout the measurement while the pulp sheet (placed on microscopic glass slide, treated with oil) was rotated for better sampling and removal of preferred orientation effects.


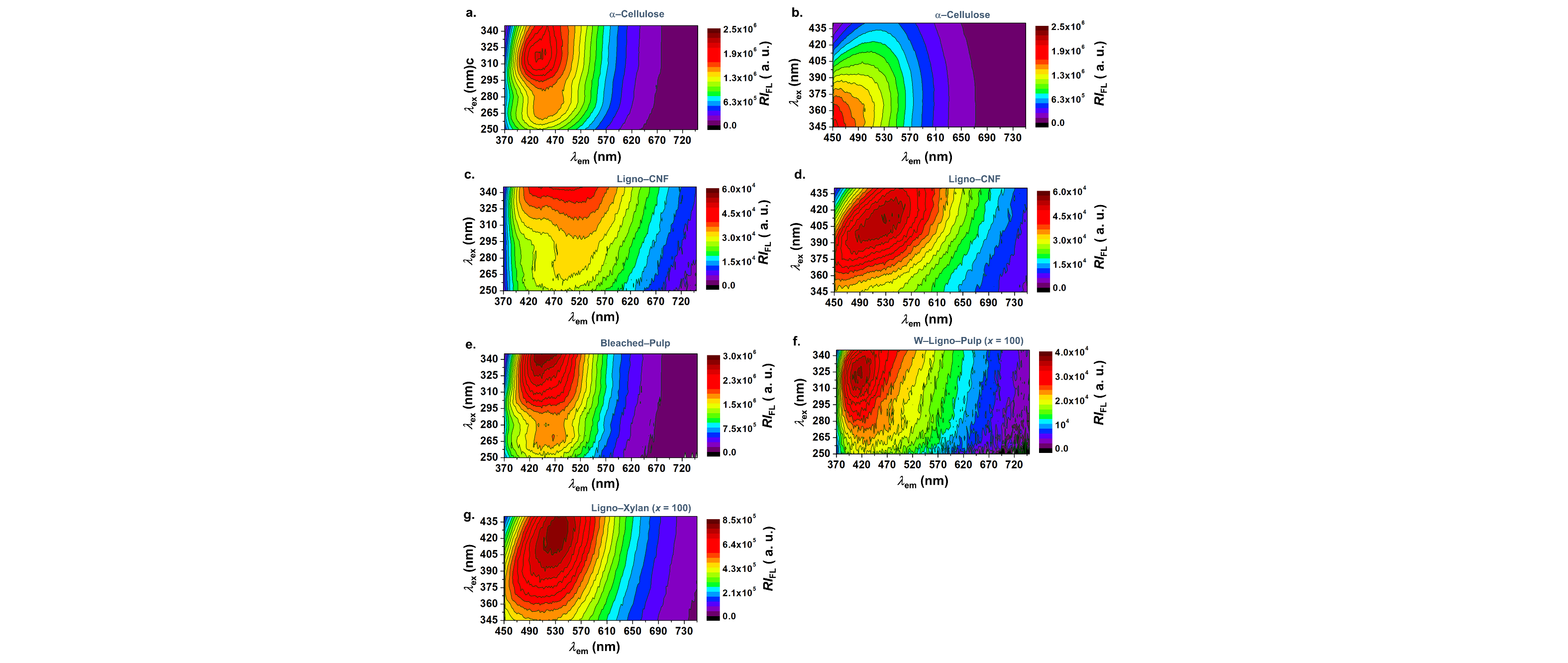


**Figure S1. EEM characterization of lignocellulosic and cellulosic materials.** 2D contour plots of (a-b) alpha-cellulose, (c-d) lignocellulosic nanofibre, (e) bleached pulp, (f) waste lignocellulosic pulp from mill, and (g) lignin containing xylan (4-*O*-Methyl-D-glucurono-D-xylan).


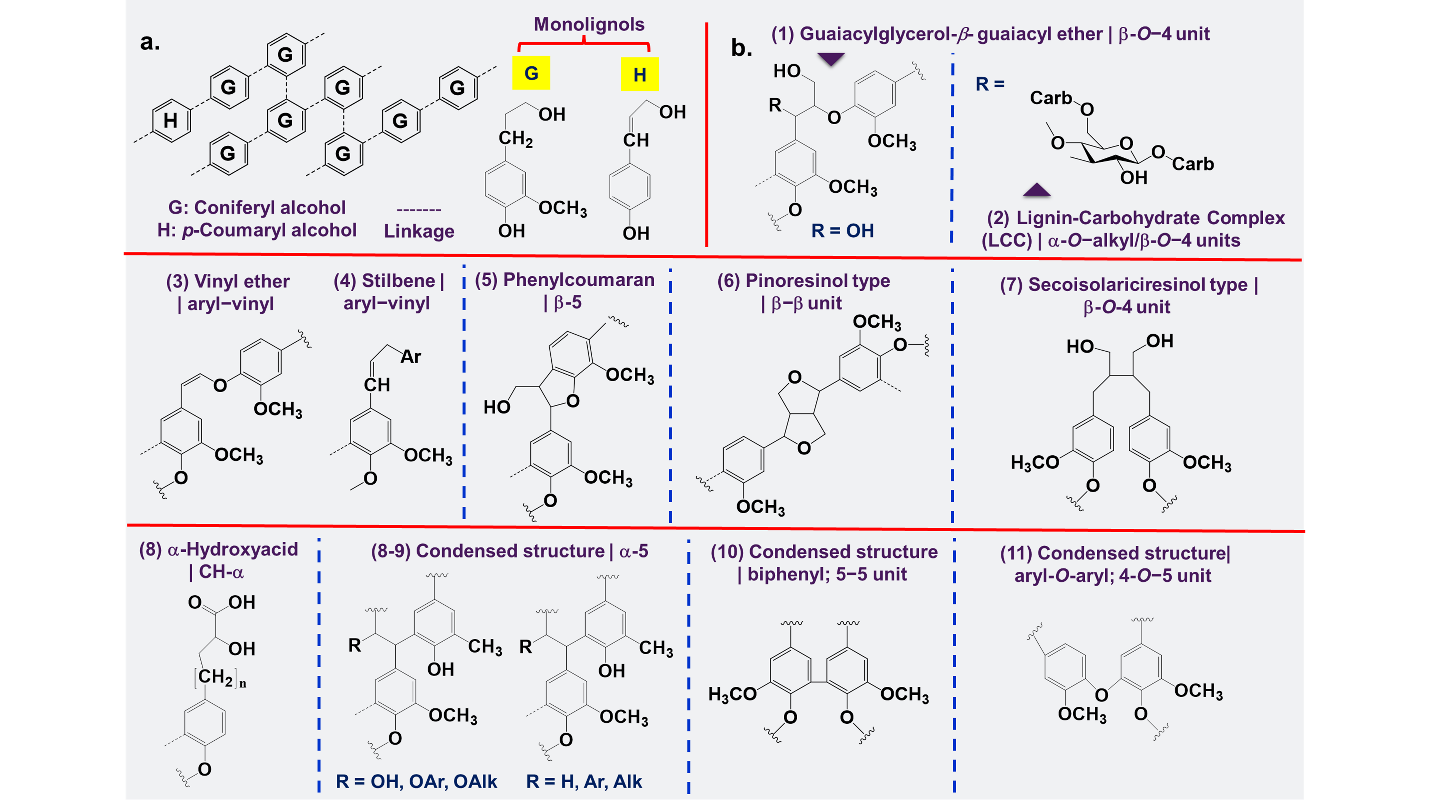


**Figure S2. Molecular structures of the softwood Kraft lignin, monomers, and inter-unit linkages.** (a) G-lignin and monomer. (b) Inter-unit linkages.


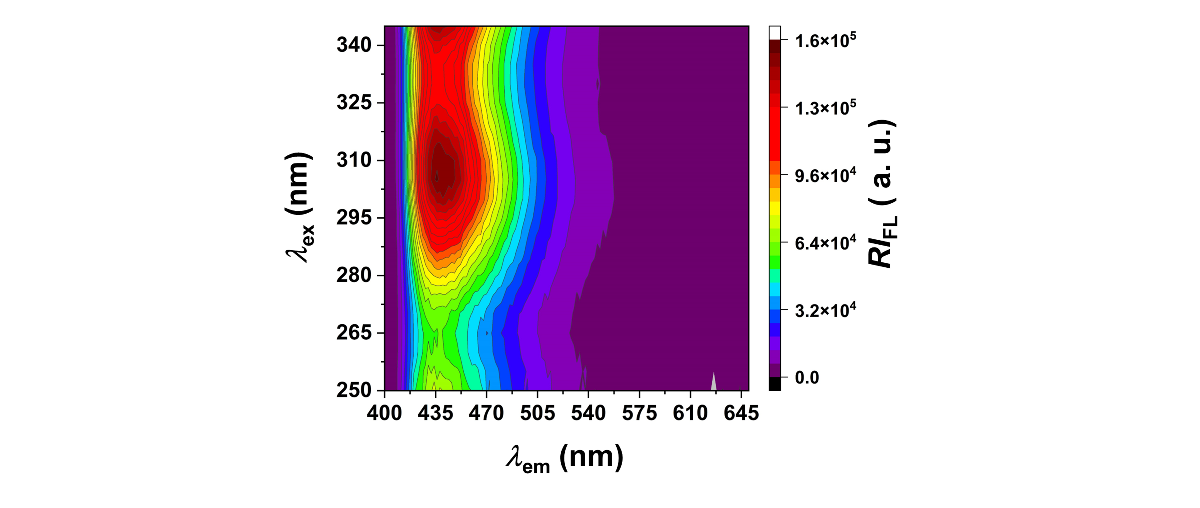


**Figure S3. EEM characterization of Evans blue synthetic dye.**


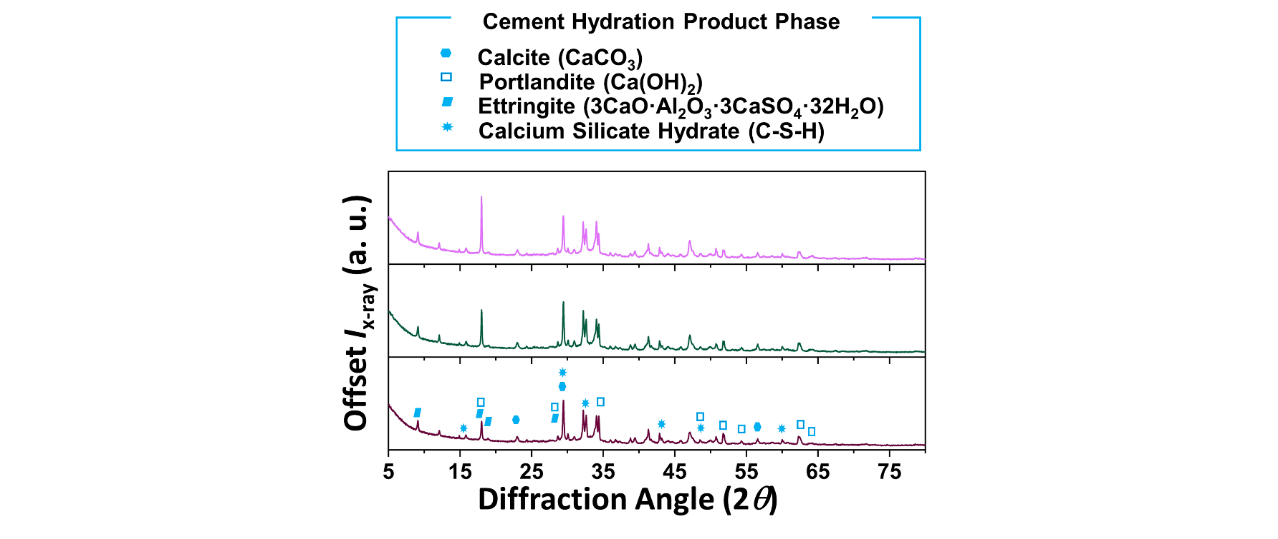


**Figure S4**. **Powder** **X-ray diffractometric (XRD) characterization of lab-made fibre cement.** Powder X-ray diffractograms of fibre cement (FC-Lab) as a function of pulp fibre content (*x* = 8, 16, and 32 wt.%). The reference patterns (powder diffraction file (PDF)) were corresponded to Calcite (01–086–2334), Portlandite (00–001–1079), Ettringite (01-075-7554), and calcium silicate hydrate (C–S–H).


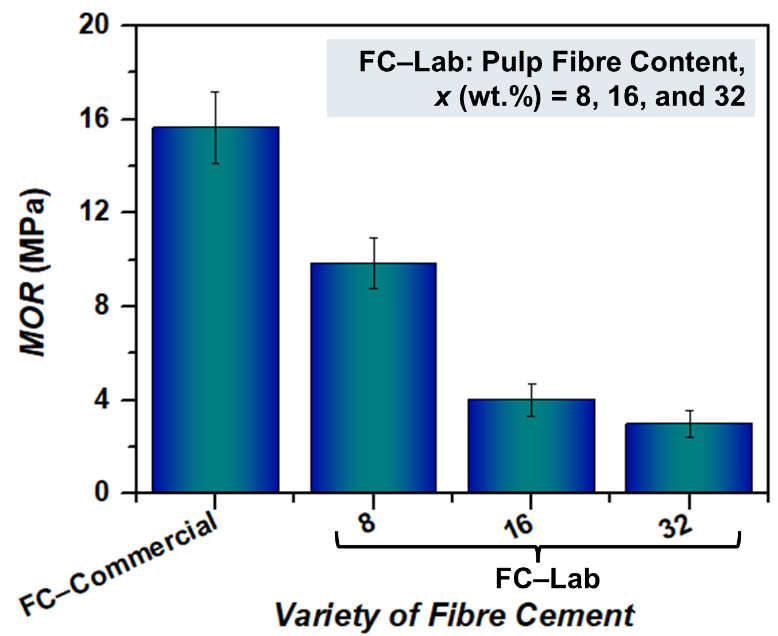


**Figure S5.** **Mechanical characterization of fibre cement.** Modulus of rupture (MOR) for lab-made and commercial fibre cement; MOR was calculated from the stress-strain curves based on the 3-point bending tests. Lab-made fibre cement samples (denoted as FC-Lab (*x* = 8, 16, 32)) were tested after 28 days. Curing time and composition of the commercial fibre cement sample (denoted as FC-Commercial) is unknown.


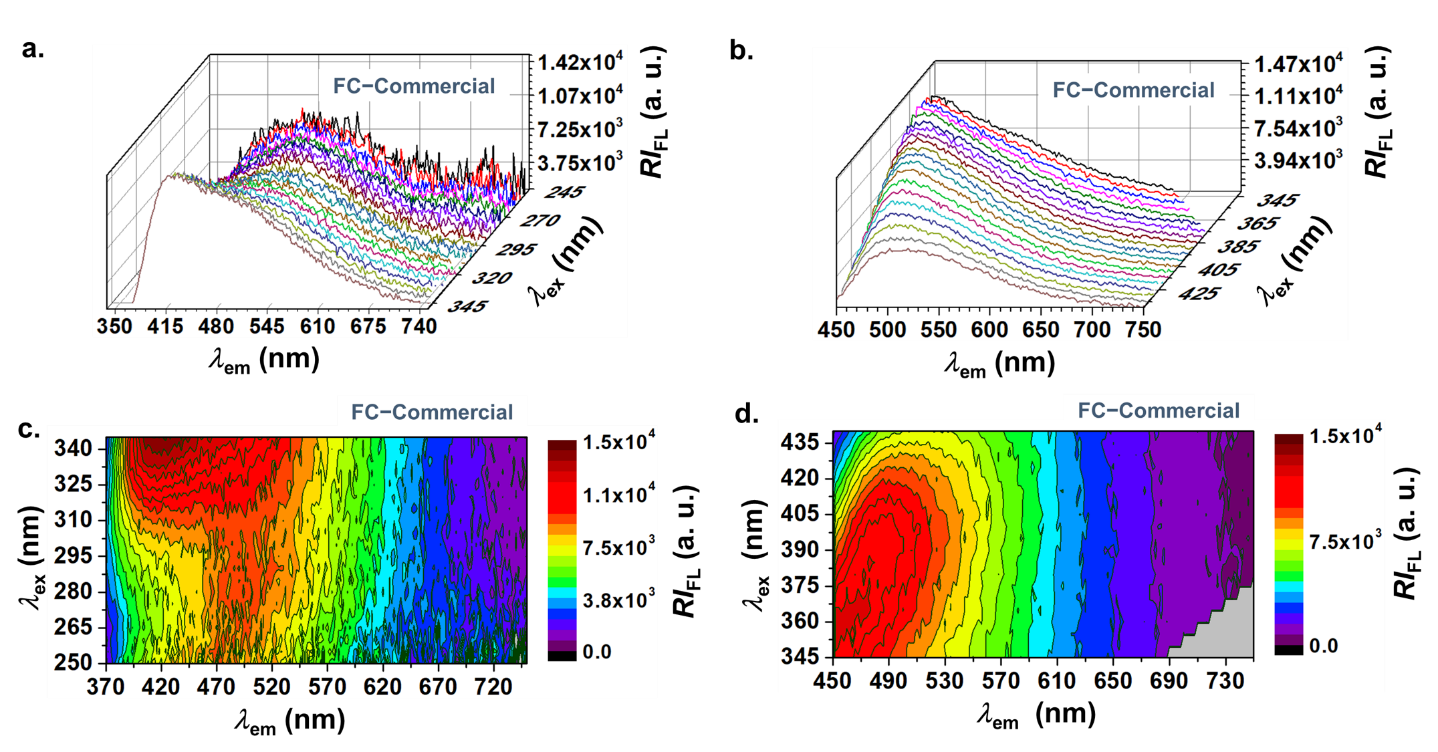
**Figure S6. EEM (emission map) characterization of commercial fibre cement.** (a-b) 3D waterfall plot (c-d) 2D contour plots of commercial fibre cement (FC-Commercial) under shorter (250–345 nm) and under longer (345–440 nm) excitation wavelength range.


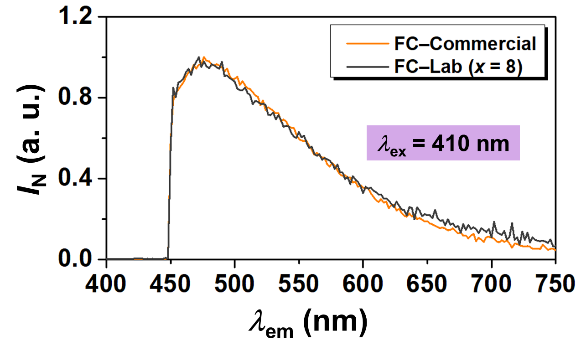


**Figure S7. Fluorometric characterization of fibre cement.** Normalized emission spectrums of lab-made (FC-Lab (*x* = 8)) and commercial fibre cement (FC-Commercial) under 410 nm excitation wavelength.

**Table S1**. Physical properties of unbleached Kraft pulp.

|  | Ligno-Pulp (K23.3) | Ligno-Pulp (K21.6) |
| --- | --- | --- |
| Conductivity (µS/cm) | 35.36 | 47.26 |
| pH | 5.28 | 5.46 |
| Fiber Length (mm) | 2.473 | 2.525 |
| Width (mm) | 28.9 | 29 |
| Aspect ratio | 85.571 | 87.069 |
| Curl | 13 | 13.4 |
| Fines (%) | 6.3 | 6.3 |


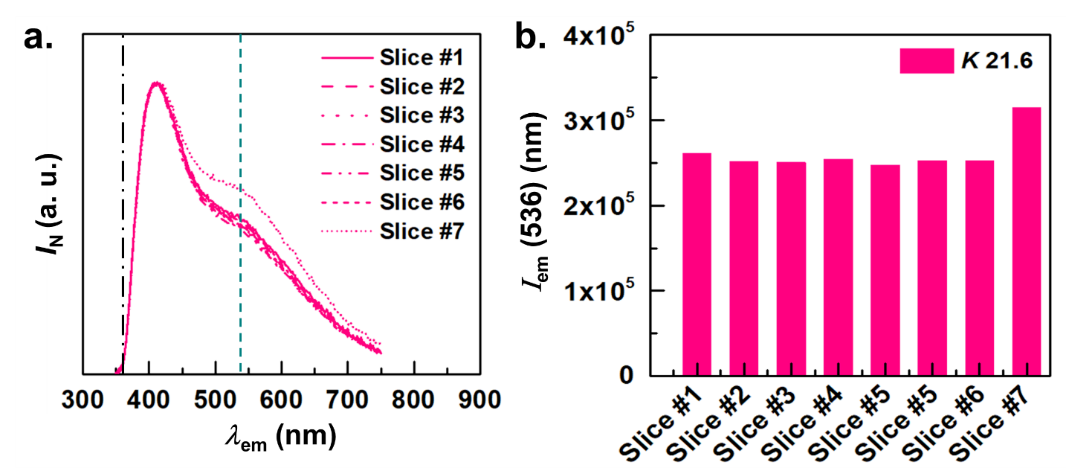


**Figure S8.** **Steady-state emission spectrum of Ligno-Pulp (*x* = 100)** (a) Emission spectrums and (b) Variation of emission intensity at 536 nm for seven different slices (1.5x1.5 cm) of pristine pulp with Kappa number (*K*) of 21.6, which were cut from the 5x5 cm pulp sheet. In **Fig. S8**a, the dotted (vertical) lines are drawn at 536 nm emission (EM) wavelength. In **Fig. S5**b, value of the EM intensity (at 536 nm) was obtained from **Fig. S8**a. All the fluorescence experiment was carried at 330 nm excitation wavelength (see, experimental section for detailed breakdown of the experimental parameters).
